# Bittersweet memories and somatic marker hypothesis: adaptive control in emotional recall facilitates long-term decision-making in the Iowa Gambling Task

**DOI:** 10.3389/fnins.2023.1214271

**Published:** 2024-01-16

**Authors:** Varsha Singh

**Affiliations:** Humanities and Social Science, Indian Institute of Technology Delhi, New Delhi, India

**Keywords:** affect regulation, emotion recall, mood, decision-making, somatic marker hypothesis, Iowa Gambling Task, affect control

## Abstract

The somatic marker hypothesis states that emotional recall and its somatic influence guide long-term decision-making. However, the mechanism through which decision-making benefits from emotional recall is unclear; whether emotional recall and the induced affect increase the regulatory demand or amplify the affect state that requires inhibition. It is unclear if controlling the automatic flow of emotion in recall improves adaptive decision-making. Two studies examine the hypothesis that affect control in emotional recall facilitates inhibitory control and benefits long-term decision-making. In Experiment 1 (*n* = 137), affect control was assessed in emotional recall to examine if switching of affect in recall of positive and negative valence (order: positive–negative memory recall vs. negative–positive memory recall) is linked with long-term decision-making. Results for long-term decision-making showed that negative–positive recall sequence was associated with higher long-term decision-making, whereas automatic frequency-based decision-making remained unaffected by the recall sequence. In experiment 2 (*n* = 71, all male), emotional recall (positive vs. negative), recall specificity (i.e., specific vs. overgeneralized recall), and post-recall mood regulation (post-recall positive mood regulation vs. no regulation) was expected to facilitate long-term decision-making. Results showed that emotional recall and post-recall mood regulation (i.e., negative recall – positive mood and positive recall - negative mood) were associated with higher long-term decision-making (decks C′ and D′). Results of frequency decision-making showed that positive emotional recall, and poor recall specificity led to infrequent punishment deck choices (decks B′ and D′). Hierarchical regression indicated that emotional recall increased infrequent deck choices and accounted for 10% of choices made, recall specificity increased the explanatory power to 19%, and higher recall specificity was associated with fewer infrequent punishment deck choices. Affect control engaged via negative emotional recall, post-recall mood regulation, and recall specificity might be a potential mechanism through which affect control in emotional recall might facilitate long-term decision-making.

## Introduction

The Iowa Gambling Task was devised to test the somatic marker hypothesis according to which impaired decision-making in ventromedial prefrontal cortex lesion patients was due to failure of somatic markers/emotions in guiding decision-making (Damasio, 1994; Bechara et al., 2000a). Emotional recall of previous choices induces a somatic state in healthy participants that helps them make long-term decisions by avoiding choices that were good for the short term but disadvantageous in the long run. The task mimics real life, that is, choosing one card from four types of card decks that vary in the outcomes: cards drawn from two decks produce large immediate rewards but carry more considerable penalties, in other words, they are risky in the long term (e.g., when chosen consistently for ten trials); whereas cards drawn from the other two decks produce small immediate rewards but result in larger rewards in the long term and are safe in the long term. As the task progresses, somatic markers (e.g., anticipatory skin conductance response) guide choices from risky decks to safe decks that produce a “net gain” across ten card picks/trials. This intertemporal decision-making involves computing long-term rewards, and shifting from prioritizing short-term to long-term rewards. In other words, the task requires shifting from the lure of immediate reward decks and the positive affect they produce to the temporary negative affect produced by foregoing the large rewards in favor of small immediate rewards that are beneficial in the long term. In real life, the choice of immediate rewards and positive affect which results in maladaptive decisions in the long term requires inhibitory control (e.g., saying no to alcohol and drugs). Intertemporal decisions in the real world rely on cognition resources such as working memory, flexibility, inhibition, and affect switching that help maintain a desired affect state, producing long-term rewards (e.g., deciding to abstain from alcohol 1 day, and each successive day could help achieve long term rewards such as regaining social/financial standing).

According to the somatic marker hypothesis, ventromedial prefrontal cortex damage patients fail to benefit from somatic markers, causing decision-making deficits, indicating that emotions (somatic markers) help in long-term decision-making (Damasio, 1994). The hypothesis attracted critical evaluations with questions related to the potential diagnostic value of the Iowa Gambling Task as a frontal lobe-based decision-making task, especially concerns related to delineating the role of executive control: that is, updating working memory, and flexibility and inhibition being raised (Dunn et al., 2006; Buelow and Suhr, 2009; Aram et al., 2019). The hypothesis lacks details in terms of how a specific somatic/affect state from the past is selected over another affect state, especially in case of mixed affect where emotional recall produced both positive and negative emotions; how does emotional recall help maintain a somatic state that can optimally bias the decision-making process (Damasio, 1994) Damasio and their associates maintain that decision-making in the task is independent of working memory, and ventromedial prefrontal cortex lesions impact decision-making tasks while dorsolateral prefrontal cortex lesions impact working memory tasks (maintenance/delay tasks), which show double dissociation (Bechara et al., 1998). However, constraints on working memory (increasing workload on the maintenance of information in the working memory) interfere with somatic markers and impair decision-making (Hinson et al., 2002), indicating that working memory might be necessary for long-term decision-making. Similarly, whether the decision-making task is independent of executive control continues to be debated (Turnbull et al., 2005; Toplak et al., 2010).

The task has a series of affect states that are evoked via varying rewards and punishments; it requires the ability to maintain reward representation, that is, drawing from safe decks that produce small immediate but large long-term rewards and, at the same time, inhibit reward drawing from risky decks that produce large immediate rewards but larger long term losses, and flexibly shift from affect states associated with immediate risky rewards to the affect states associated with delayed, safe, long term rewards (e.g., emotional recall of alcohol-laced parties could induce a positive affect, when regulated and turned to emotional recall of the following hangover and negative affect can help make long-term decisions to abstain from alcohol). Despite the demands on affect control in the form of the ability to switch, inhibit, and shift between positive and negative affect, the role of affect control in somatic marker hypothesis and task decision-making remains unexplored. It is possible that the affect induced in emotional recall requires inhibition and regulatory control to make an adaptive decision (e.g., negative emotional recall of a failed relationship could heighten social anxiety, if unregulated, and it might be detrimental to seeking social support). Recently, affect control has been considered as the use of cognitive control in an affect context (Schweizer et al., 2020). When affect-incongruence of emotional recall and decision-making is created via experimental material (e.g., a congruent affect state was induced by presenting rewards with a positive word, and an incongruent state was produced by presenting rewards with a negative word), it depleted cognitive resources such as working memory and was detrimental to somatic markers and decision-making (Hinson et al., 2006). On the other hand, affect control in emotional recall is an essential mechanism of emotion self-regulation; negative affect in healthy people is ameliorated via mood-incongruent recall of positive autobiographical memories that inhibit negative affect, which switches the prevailing negative affect to a positive affect (Parrot and Spackman, 2000; Cooney et al., 2007). Affect control in emotional recall might be critical for self-regulation and adaptive long-term decision-making.

According to the somatic marker hypothesis, emotional recall biases choices toward long-term decision-making; however, unregulated affect in emotional recall might interfere with cognitive resources that are necessary for long-term decision-making, as observed when self-regulation is absent, such as in depression, where emotional recall of positive emotion to repair negative mood is absent (Joormann and Stanton, 2016). Surprisingly, no study has examined the regulatory control of affect (i.e., affect control) in emotional recall on decision-making in the Iowa Gambling Task. Previous efforts to understand the impact of emotional recall on long-term decision-making showed that the affect induced by emotional recall (e.g., emotional recall in response to category cues such as happiness, sadness, fear, and anger) did not improve long-term decision-making (Bechara et al., 2000b). The interference was detrimental to long-term decision-making (Bechara and Damasio, 2005). Affect control (inhibition and switching between polarized affect states such as negative affect to positive affect) is implicated in long-term decision-making because it entails attention shifting away from the positive affect of the previously rewarded options to the negative affect of foregoing rewards; this inhibitory control is critical for long term decision making (Fellows and Farah, 2005). The mechanism through which emotional recall may facilitate long-term decision-making is poorly understood (Dunn et al., 2006), possibly because the role of affect control in emotional recall (use of executive processes in controlling affect in emotional recall) has not been explored within the context of the hypothesis and the task performance. Control over the affect induced via emotional recall is a potential mechanism through which emotions might improve, inhibiting risky, impulsive decisions and leading to safe long-term decisions. In specifying emotional recall, studies have focused on working memory paradigms using emotion-eliciting words as cues, emotional pictures, sounds, and other experimental material (e.g., Bechara et al., 2000a; Hinson et al., 2002, 2006; Brevers et al., 2015). The present study employed a self-related memory system of autobiographical episodic recall because it engages autonoetic consciousness that allows one to examine recall of the time and place of personal emotion-involving events; the recall relies on going back in time to re-experience the event (Wheeler et al., 1997; Tulving, 2002). Although both autobiographical and episodic recall engage the prefrontal cortex circuitry (Gilboa, 2004), overlap between recall of episodic memory and the Iowa gambling task has been explored (Turnbull et al., 2014).

Unlike other declarative memory (e.g., semantic words, pictures), autobiographical episodic recall reflects the autonoetic experience associated with imagining events in the past and future (D'Argembeau et al., 2008); unlike emotional recall of experimental stimuli (words, sounds, pictures), this self-referential recall might reflect the information processing framework of a “body loop” (original emotion experience) and “as if body loop” (recalled emotion experience) described in the somatic marker hypothesis (Damasio, 1994, p. 156). Because the somatic marker hypothesis relies on inducting somatic influences from the recall of significant life events, using the autobiographical episodic recall of previous life-altering decisions might be well suited for understanding the link between affect control in emotional recall and long-term decision-making. Emotional recall of significant decisions (e.g., remembering a life-defining career decision such as choosing between a career that is closer to one's interests and desires vs. one that provides social status and financial security) will entail searching, sorting, switching, and alternating between positive and negative affect states evoked by the emotional recall of that decision (e.g., pride/joy of opting for a career track that one likes vs. the worry/sadness of giving of up lucrative career option). When emotional recall is accompanied by affect control, it will enable efficient switching between positive and negative affect states evoked by the emotional recall to select specific affect associated with the decision to be re-experienced (e.g., an overall joy or overall worry associated with the decision). Because emotional recall re-produces the affective state associated with the past event, affect control in emotional recall might indicate a successful shift between affect states. Repeating the emotional recall via sequential emotional recall of two polarized emotions (e.g., positive emotional recall followed by negative emotional recall), affect control will be reflected in the ability to flexibly inhibit and switch between affect states (e.g., positive emotional recall associated with re-experiencing positive affect will be followed by negative emotional recall associated with re-experiencing negative affect). Therefore, sequential emotional recall will entail affect control, that is, inhibition and switching between one affect to another affect (from the first to the second emotional recall).

It is proposed that affect control (use of executive control in affect context) might be a mechanism through which emotional recall might facilitate long-term decision-making. Affect control might facilitate inhibitory control required for switching from short-term rewards toward long-term rewards via inhibiting and flexibly switching between affect states in emotional recall and affect states evoked by rewards and punishments in the decision-making task. It was hypothesized that affect control in emotional recall will facilitate cognition-intensive long-term decision-making. In contrast, affect control will not benefit frequency-based decision-making because it engages fewer cognitive resources. Affect control and long-term decision-making in the Iowa Gambling Task were examined in two experiments. In Experiment 1, it was expected that the two emotional recall sequences (negative–positive recall vs. positive–negative recall) would evoke affect-switching between positive and negative affect and be linked with long-term decision-making. In Experiment 2, it was expected that affect switching enables recall specificity (specificity in negative vs. positive recall) and post-recall mood regulation (negative recall–positive mood shift vs. positive recall–negative mood) and will be linked with long-term decision-making. Affect control in emotional recall, recall specificity, and post-recall mood was expected to be linked with long-term decision-making, specifically in the case of negative emotion. It is essential to specify that the present work did not aim to examine the relationship between emotional recall (autobiographical episodic) and decision-making, but whether affect control in emotional recall (inhibiting, switching between affect) facilitates long-term decision-making in the task was of interest.

Further, it remains unclear why healthy participants in the studies on the Iowa Gambling Task choose reward-punishment frequency over long-term decision-making (i.e., choosing decks based on frequent vs. infrequent punishments) (Lin et al., 2007). Frequency-based decisions are defined as when decision makers choose the decks that yield frequent rewards vs. those that give infrequent punishments/losses (e.g., drawing ten cards from a frequent reward deck might give five small rewards when chosen in ten trials vs. infrequently rewarding deck will give one large reward when chosen in ten trials). Frequency-based choices reflect fewer cognitive resources such as working memory and are considered unregulated/automatic, emotion-based processing that shows double dissociation from cognitively demanding, long-term decision-making (Singh and Khan, 2009, 2012; Stocco et al., 2009; Singh, 2013b). In line with the dual-process account of cognition and emotion-based information processing (e.g., Tversky and Kahneman, 1971; Evans, 2003), affect control might not be linked with long-term decision-making but also might not impact frequency-based decision-making. The approach in experiment 1 was to generate affect control via two types of emotional recall sequence that counterbalanced positive and negative emotional recall; the approach in experiment 2 was to examine affect control in two types of recall (positive vs. negative emotional recall), two types of recall specificity (specific vs. overgeneralized), and two types of post-recall mood regulation (post-recall positive regulation vs. non-regulation). These approaches explored whether affect control in emotional recall might facilitate long-term decision-making in the Iowa Gambling Task.

## Experiment 1

Affect control in alternation, shifting between affect and emotional recall, might vary in cognitive demands in a valence-specific manner. For instance, disengaging/switching from negative emotional recall or affect shift from negative to positive affect might be more demanding because negative recall has more robust affective, cognitive, and physiological responses than positive or neutral experiences (Taylor, 1991), and information conveyed by negative experience is priority-processed (Fiske, 1980; Peeters and Czapinski, 1990; Mogg and Bradley, 1998; Cacioppo and Gardner, 1999). Negative experiences are easy to remember and recall (Morewedge et al., 2005), are more likely to be retrieved while anticipating future outcomes, and disproportionately influence decision-making (Tversky and Kahneman, 1973; Morewedge et al., 2005). Neural circuitry associated with long-term decision-making (Li et al., 2010) and affect regulation (Maratos et al., 2001; Ochsner and Gross, 2005) shows reduced activation when negative emotions are recalled (Damasio et al., 2000), indicating that switching from negative affect might be demanding. Because negative recall might have an asymmetrical influence, it was expected that the sequence of negative recall followed by positive recall would recruit more significant effort in affect switching and regulation (disengaging and switching from negative emotional recall) and will be associated with long-term decision-making. Because recall of the outcomes associated with the previous choice/decks is required for shifting choices from decks that are rewarding in the short-term towards decks that are rewarding in the long-term, it was believed that the emotional recall sequence would recruit affect control and hence impact long-term decision making, but frequency-based decision-making will not benefit from affect control evoked by the sequence of emotional recall.

### Method

#### Participants

A power analysis (G power) was done to determine a sample size that would be sufficient to reach the desired power (0.95) and small effect size (0.40), and this was found to be 86. One hundred and thirty-seven undergraduate and graduate students volunteered for the study in two phases. Forty-five undergraduate and graduate students volunteered in the first phase (mean age = 24.93 years, SD = 2.48; 24 male), and ninety-two participants volunteered in the second phase (mean age = 22.85 years, SD = 3.04; 46 male). Participant recruitment took place in two phases due to student availability for research participation (an intervening summer vacation between two semesters, when students are expected to vacate the campus).

#### Materials

##### Emotional recall

Participants were asked to recall their earliest and most significant emotion-evoking experience for positive emotion, an experience that resulted in a positive outcome and induced positive emotions; and likewise for negative emotion, an experience resulting in a negative outcome and that induced negative emotions. All participants recalled positive and negative emotional decisions in one of the two sequences: negative emotional recall followed by positive emotional recall, or the reverse order. Participants were instructed to write and describe the emotions experienced at that time on a sheet of paper with no specified time limit. Writing about autobiographical recall is a valid measure of inducing emotion experienced at the time of the event that the emotions were experienced (Mills and D'Mello, 2014).

Further, participants were encouraged to report the earliest memory of emotional decision because the recall of older memories generates more significant psychophysiological responses due to frequent activation (Foster and Webster, 2001), and were advised to recall the two emotional events from a similar period, with a maximum of a year apart. In addition to inducing emotions through writing, a self-rating of recall strength was obtained for both positive and negative recall; it was expected that the ability to recall emotion would produce a higher self-rating for the strength of the emotional recall. Three statements were used to assess the strength of the emotional recall on a 5-point scale (*strongly disagree* to *agree strongly*). The statements used were as follows: (1) “I often think about this decision,” (2) “I remember every detail of this decision very well,” and (3) “I clearly remember the circumstances in which this decision was made.” The strength of self-rated emotional recall ranged from 3 to 15 for each recall session, with 15 indicating the maximum strength for each recall. Memories depicted in emotional recall represented student life; examples of positive emotional recall included passing the rigorous entrance exam for an elite engineering program or achieving their chosen career stream. Examples of emotional recall of negative valence were the experience of yielding to family pressure when deciding on a specialization, choosing the wrong career track, experiencing homesickness on leaving home, or the dissolution of a failed relationship.

##### Iowa Gambling Task

The decision-making task employed in the study was the computerized Iowa Gambling Task (IGT: Bechara et al., 2000a). The participant began the task with 2000 play points and was instructed to maximize gains and minimize losses (see Appendix A for complete task instructions). In order to ensure task motivation, participants were asked to estimate the number of points that they thought they would win in the game (range: 0–10,000) before the task started (“Based on your judgment, please give an estimate ranging from 0–10,000 to indicate how many points do you plan to win in this game?”). Since self-set goals determine the cognitive resources that will be committed to decision-making (Schiebener et al., 2012, 2014), even implicit goals drive cognition-intensive intertemporal decision-making (Hassin et al., 2009), it was assumed that this estimate would enable a self-set goal and motivated decision-making in the task. They were then instructed to pick one card at a time from the four decks of cards labeled A', B', C', and D'. When a card was drawn, the amount of play money “won” was announced, and at times, this announcement was of a “loss.” Participants were further told that sometimes the cards would result in a loss and that they should try to make more profit and stay away from the cards that result in a loss. They were then asked to continue making choices until the game ended. Participants were unaware of the number of choices to make (i.e., 100 card picks) and the decks' reward-punishment schedule. The reward and punishment schedule of the four decks differed along two attributes: (a) intertemporal—decks A′ and B′ (risky decks) had high immediate rewards and resulted in a net loss in the long-term, vs. decks C′ and D′ (safe decks) which had low immediate rewards and resulted in a net gain, (b) frequency—decks B′ and D′ had infrequent punishment (used henceforth as infrequent punishment decks) vs. decks A′ and C′ had frequent punishment. The choice of infrequent punishment decks (B′ and D′) reflected a preference for low-frequency punishment.

In contrast, the choice of decks A′ and C′ reflected tolerance of high-frequency punishment/low-frequency rewards. As mentioned in the Introduction, these two attributes of decision-making reflect two ways risk is perceived in the IGT (Singh, 2013a), such that decisions made based on the intertemporal attribute reflect cognition-intensive risk processing. In contrast, those made based on the frequency attribute reflect cognition-independent automatic risk processing (Singh, 2013b).

#### Procedure

Participants reported to the laboratory between 9:00 AM and 12:00 PM and gave informed consent after receiving the study details. The Institute Ethical Committee approved the protocol. Participants recalled memories of two emotional decisions in one of two orders –the decision with positive outcomes, followed by one with adverse outcomes, or the reverse. In the first phase, all 45 participants recalled positive followed by negative. In the second phase, odd and even numbers were used to assign the 92 participants to one of the orders (negative-positive or positive-negative, 46 participants each). Participants wrote a detailed description of the experience of how they felt at that time and were asked to rate that recall. The participants were given one blank double-sided A4 size sheet of paper to write the description, with no time limit for the writing, and a 5-min break was given between the two emotional recall sessions. The emotional recall session was followed by a short break (10 min) followed by the decision-making task. Challenges have been noted in the use of experimental procedures to test the effect of emotional recall (autobiographical episodic recall) and affect induction, for instance, complexity in counterbalancing of recall, and requiring an uninstructed long recovery period to ensure reduced carryover effects from previous emotional recall sessions (Gillihan et al., 2011). The task instructions were read out to participants before administering the IGT. After ensuring participants understood the task instructions, they were asked to clarify any doubts. After completing the task, the participants were debriefed and thanked for participating.

#### Data analysis

A paired *t*-test showed that self-rating of emotional recall for experiences of positive and negative emotions did not differ in phase one of the data collection, which had participants' emotional recall order as positive recall followed by negative t_(44)_ = −1.58, *p* = 0.12. In phase two of the data collection (half the subjects followed a recall order of positive-negative and the other half followed a sequence of negative-positive recall) there was no difference in self-rating recall of positive and negative emotional recall t_(91)_ = 0.71, *p* = 0.45. Because participants recruited in the two phases showed no differences in the recall sequence, responses were merged in one dataset. For the first analysis, cognition-intensive intertemporal decision-making was defined as the number of cards drawn from decks C′ and D′, the long-term safe decks. The mixed model ANOVA addressed emotional recall 2 (emotional recall sequence: positive–negative recall vs. negative-positive recall) × 2 (intertemporal choices: safe decks C′ + D′ vs. risky choices A′ + B′) was examined (Type II sum of squares, correcting for an unbalanced group, Langsrud, 2003). Emotional recall sequence was a between-group variable, and deck choices were within-group variables. Because age influences task decision-making (Beitz et al., 2014) and performance shows male advantage (Singh, 2016; Singh et al., 2020), median-coded age and gender were covariates. The second analysis addressed the effect of emotional recall sequence and covariates on frequency-based decision-making defined as a preference for infrequent punishment decks (i.e., the number of cards drawn from decks B′ and D′) in a 2 (recall: positive-negative vs. negative-positive) × 2 (frequency: frequent punishment decks A′ + C′ vs. infrequent punishment decks B′ + D′). Data analysis was performed using the Statistical Package for Social Sciences (16), with the level of significance set to 0.05.

### Results

The results showed a significant effect of emotional recall sequence *F*_(1, 133)_ = 4.56, *p* = 0.03, $\eta_{\text{p}}^{2}$ = 0.03, suggesting that participants who recalled negative emotion followed by positive emotion made more long-term advantageous choices (*M* = 64.25) compared to those who first recalled positive emotion (*M* = 55. 97). The covariates had no effect (*p* > 0.05 for age and gender).

The results for frequency-based decision-making (choice of cards from infrequent punishment decks B′ and D′) indicated that recall sequence did not affect frequency-based decision-making [*F*_(1, 133)_ = 1.56, *p* = 0.21], preference for infrequent punishments remained the same in negative-positive valence recall sequence (*M* = 56.56) and positive-negative recall sequence (*M* = 59.23). The covariates had no effect (*p* > 0.05 for age and gender).

**Figure 1:**
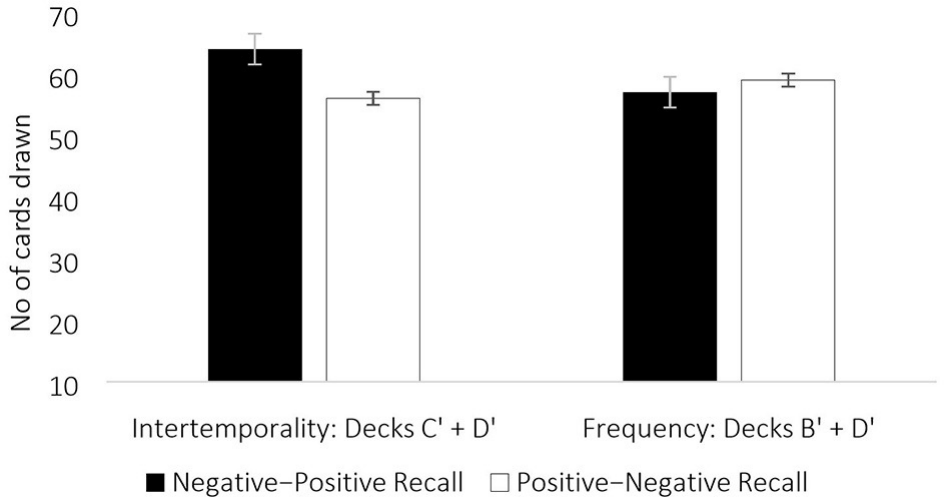
Interaction between the emotional recall sequence and long-term decks (C' +D') was significant [*F*_(1, 133)_ = 4.56, *p* = 0.03, $\eta_{\text{p}}^{2}$ = 0.03], whereas frequency-based choices remained indifferent to emotional recall sequence [*F*_(1, 133)_ = 1.56, *p* = 0.21]. Error bars indicate standard errors.

Results suggest that affect control elicited via negative-positive recall sequence compared to positive-negative recall was associated with cognition-intensive intertemporal decision making, indicating that regulatory control in disengaging from negative emotional recall was associated with more long-term decision making. Affect control in sequenced emotional recall was not linked with frequency-based decision-making, indicating that regulatory demand might not influence the automatic processing of frequency attributes in decision-making (Figure 1).

### Discussion

The effect of emotional recall sequence (negative–positive recall vs. positive–negative recall) was examined for intertemporal decision-making (short-term decks A' + B' vs. long-term decks C' + D') and frequency-based decision-making (frequent punishment decks A' + C' vs. infrequent punishment decks B' + D'). As expected, emotional recall sequence influenced long-term decision making, specifically negative–positive recall sequence was associated with long-term decision making such that more choices were made from safe decks rather than risky decks. Recall sequence (negative-positive vs. positive-negative) entailed shifting switching between recall of two polarized states of negative–positive affect, and if treated as two separate tasks with a separate goal (recall of a negative event and recall of a positive event), having to switch between two tasks from the first emotional recall to the second emotional recall entails shifting, updating and enabling goals (Meiran et al., 2000). It might recruit executive functions of shifting, updating, and inhibiting (Miyake et al., 2000) in the context of affect control in emotional recall (Schweizer et al., 2020). This explanation aligns with affect regulation, where switching and inhibitory control of affect in emotional recall facilitates emotion regulation (Ochsner and Gross, 2005). Because long-term choices rely on shifting attention from immediate rewards to delayed rewards, updating the intertemporal reward information and inhibiting the selection of short-term rewards is enabled by enabling control, switching, and inhibition of affect states. In line with the dual process account, emotional recall sequence influenced cognition-intensive long-term decision-making, but automatic, emotion-based processing of frequency-based decision-making showed no effect of emotional recall sequence.

As expected, negative—positive recall sequence was associated with more long-term choices than positive—negative emotional recall. It was believed that the negative-positive recall sequence would require more cognitive resources for disengaging from negative affect induced by negative emotional recall and switching to positive emotional recall and inducing positive affect. Studies have documented the ‘negativity bias' which is a disproportionate impact of negative emotional recall; for instance, negative information demands more cognitive resources (Schwarz, 1991; Taylor, 1991), and therefore switching from negative affect demands greater regulatory control (Ochsner and Gross, 2005). The emotional recall sequence might require a negative emotional recall to be followed by a positive emotional recall; it might be accomplished with greater inhibitory control, and the inhibitory control required for making long-term advantageous cards might be facilitated by higher affect control as compared to the affect control engaged by positive recall preceding negative recall. The results indicate that affect control in emotional recall where negative emotional recall preceded positive emotional recall was linked with better inhibitory control and long-term decision making (compared to recall sequence where positive emotional recall precedes negative emotional recall); the result is in line with other observations where the induction of negative mood shows improvement in long-term choices in the task (Buelow and Suhr, 2013). Others have observed that switching from negative affect improves working memory capacity (Schmeichel and Demaree, 2010) and improves long-term decision-making (Heilman et al., 2010; Bollon and Bagneux, 2013). Greater affect control (engaged by negative–positive emotional recall) might be associated with more cognition-intensive long-term decision-making, which relies on deliberate, reflective, and controlled information processing. Compared to affect control deployed in switching from negative to positive emotional recall, the recall sequence of positive to negative emotional recall engages less affect control, and potentially the reason why fewer choices from long-term reward decks were associated with the positive-negative recall; it might indicate that the greater the affect control in emotional recall, the better the long term decision making. Aligned with the dual-process accounts, this resource-dependent affect control (cognitive resources such as switching, flexibility, and inhibition required for affect control in emotional recall) might be further substantiated by the results where frequency-based decision-making remained unaffected by the recall sequence.

## Experiment 2

Disengaging from negative emotional recall in the negative-positive sequence required greater affect control, potentially enabling long-term decision-making. It indicates that affect control in emotional recall facilitates long-term decision-making in the Iowa Gambling Task, where frequency-based decision-making is unaffected by emotional recall order. Sequential emotional recall of negative and positive emotions was presumably enabled via flexibly inhibiting and switching between two polarized affect states evoked by two emotional recall sequences. The present efforts aim to identify how affect control in single-emotional recall impacts long-term decision-making. It was proposed that affect control manifests in recall specificity and post-recall mood regulation.

When an episodic event is recalled (positive or negative), affect control in emotional recall can be understood in two ways—first by examining the specificity of emotional recall (Williams et al., 2007), when recall shows high specificity (i.e., when precise details of time, place of the event was recalled as opposed to when recall produces an overgeneralized, expansive, or a non-specific recall in terms of retrieving memory of a single instance occurring in a specific time and place). Higher specificity of emotional recall is associated with higher cognitive control and accurate imagining of future events; presumably, the prior facilitates the latter (Williams et al., 1996, 2007). Emotional recall entails an effortful search for details related to the event; retrieval of specific events and time requires cognitive resources such as inhibiting and flexibly switching between events and affect states, limiting unnecessary and/or unrelated details. Consequently, affect control will be reflected in high recall specificity in emotional recall, and it might facilitate inhibitory control required for long-term decision-making. Further, it is observed that poor recall specificity, especially in negative recall, reflects poor affect control or poor usage of cognitive control in affective experience (Conway and Pleydell-Pearce, 2000), especially in affective disorders such as depression, which is characterized by negative affect (Dalgleish et al., 2008).

Additionally, recent studies implicate ventromedial prefrontal cortex in recall of negative affect experienced in the past month (Zald et al., 2002), negative affect engages greater cognitive control (Ochsner and Gross, 2005) the negative–positive affect implicates the subregion of ventromedial and dorsolateral prefrontal cortex in depression, a disorder characteristic of negative affect (Northoff et al., 2000; Koenigs and Grafman, 2009). Therefore, negative emotional recall (vs. positive emotional recall) in the presence of recall specificity (compared to the absence of specificity) was expected to reflect high affect control in emotional recall and facilitate long-term decision-making in the Iowa gambling task.

The second way to examine affect control in emotional recall is via post-recall affect regulation. When emotional recall induces a memory-congruent mood, negative recall induces a negative mood, and a positive recall will induce a positive mood, autobiographical recall is a reliable method of mood repair or induction (Mills and D'Mello, 2014). It is argued that self-regulation in negative affect via mood-incongruent recall of positive autobiographical memories enables control of negative affect and flexibe switching from negative affect to positive affect induced by positive recall (Parrot and Spackman, 2000; Cooney et al., 2007). The incongruent post-recall mood reflects affect switching, with more resources required for negative emotion recall being repaired by post-recall mood shift from negative to positive affect, as switching away from negative affect demands greater regulatory control (Ochsner and Gross, 2005). Conversely, failure of the post-recall mood switch will reflect poor affect control. Affect control in emotional recall in two forms (recall specificity and post-recall mood regulation) was expected to be linked with cognitive-intensive long-term decision-making.

### Method

#### Participants

Eighty-five undergraduate and graduate students responded to a call for participation in an emotion and decision-making study and volunteered to participate. Due to fewer female participants (males = 72; females = 13), the analysis was carried out on an all-male sample (mean age = 20.31 years, SD = 1.10) with data missing for one participant (*N* = 71).

#### Materials

The emotional recall procedure and Gambling Task were identical to Experiment 1, except that emotional recall was done for a single valence, and the recall description was analyzed to examine whether the recall's nature showed specificity or over-generalization. The autobiographical memory task criterion (Williams et al., 1996) was used to analyze specificity in valence recall; as per the criterion, the recalled description was considered specific when the decision event described had taken place within a particular time, on a specific day, and did not last for more than a day.

Positive and negative affect schedule

PANAS (Watson et al., 1988) consists of 20 items that assess positive (e.g., interested and excited) and negative affect (e.g., nervous and afraid) and was used to assess post-recall mood regulation. Cronbach's alpha was acceptable for the ten positive and negative affect items (>0.80).

#### Procedure

Participants were given an overview of the study and provided informed consent. The Institutional Ethics Committee (IEC) approved the procedure. Participants were assigned to one of the two conditions of recall (positive recall or negative recall) using odd-even numbers to the order in which the participants enrolled in the study. The procedure for the emotional recall task was the same as in study 1 (i.e., provide a written description of an emotion-involving decision followed by a subjective rating of that recall), except for half of the participants recalled a positive emotion (*n* = 32, excluding one missing data) and the other half recalled a negative emotion (*n* = 38). The written description and the self-rated strength of the recall were to facilitate recall with appropriate valence, negative or positive, as per the participant's subjective judgment. Participants answered the PANAS questionnaire after they completed the emotional recall session. The emotional recall and mood questionnaire were followed by a short break (10 min) and the decision-making task. The task instructions were read out to participants before administering the IGT after ensuring that the participants understood the task instructions, like in Experiment 1. After completing the task, the participants were debriefed and thanked for participating.

#### Data analysis

Mixed ANOVA examined emotional recall, recall specificity, and post-recall mood on intertemporal deck choices as within-subject variables. The analysis was repeated for frequency-based choices as within-subject variables. The effect of emotional recall enabled a comparison between positive and negative recall. The two recall groups were positive (*n* = 33, after one participant with missing data was excluded) vs. negative recall (*n* = 38) and showed no significant difference in the strength of recall, indicating that both the groups could recall and were equally successful in recall (a most significant decision that involved emotions). For recall specificity, criterion-based coding of the recall was used to group participants based on recall specificity separately for positive recall and negative recall: participants with recall specificity (*n* = 37) and those whose recall showed over-generalization (*n* = 34). The post-recall mood was coded to reflect the positive mood on intertemporal decision-making. The ratio of total positive mood scores to the total negative mood scores indicated a prevalence of positive mood, and a median-based cut-off (median = 2.17) was used to group participants based on those who showed positive post-recall mood regulation (*n* = 36) and those who did not show positive post-recall mood regulation (*n* = 35). In both analyses, emotional recall (positive recall vs. negative recall), recall specificity (specific vs. over-generalized) and post-recall mood regulation (positive regulation vs. non-regulation) served as between-subject variables. Cognition-intensive, intertemporal decision-making was defined by the number of cards chosen from the long-term decks (i.e., the number of cards drawn from decks C′ and D′ vs. those drawn from decks A′ and B′). Similar analyses were carried out for frequency-based decision-making, defined as number of cards drawn from the infrequent punishment decks (i.e., the number of cards drawn from decks B′ and D′ vs. those drawn from decks A′ and C′). It was expected that negative emotional recall in the presence of recall specificity and post-recall mood regulation would be associated with higher long-term choices in the task.

In contrast, frequency-based decision-making will be associated with positive emotional recall, poor recall specificity, and poor post-recall mood regulation. While a mixed ANOVA offered insight into deck choices made by the groups, carrying out additional hierarchical regression offered support. It enabled comparison by adding variables to compare how emotional recall, recall specificity, and post-recall mood might account for decision-making. Data analysis was performed using the Statistical Package for Social Sciences (SPSS 16), with the level of significance set to 0.05.

### Results

One-way ANOVA with emotion recall as a between-subject variable (positive vs. negative) showed no significant difference between the subjective ratings of the strength of emotional recall, suggesting that both the groups' recall had a similar strength of recall, indicating memorability (most significant emotion involving decision).

The effects of emotional recall, recall specificity, and post-recall mood regulation were examined on long-term choices. The results for long-term decision-making showed the main effect of long-term deck choice was significant; participants made more long-term choices (M = 56.68, 95% CI 53.48–59.87) compared to short-term choices (M = 43.32, 95% CI 40.13–46.52). However, only the interaction of emotional recall and the post-recall mood was significant; negative emotional recall and post-recall positive mood modulation showed more long-term deck choices (M = 61.18, 95% CI 55.36–67.01) than short-term decision choices (M = 38.82, 95% CI 32.99–44.64) compared to negative recall and absence of post-recall positive mood showing undifferentiated long term (M = 52.99, 95% CI 46.69–59.29) vs. short deck choices (M = 47.01, 95% CI 40.71–53.31), or positive emotional recall and negative post-recall mood modulation that showed more long term compared (M = 60.44, 95% CI 54.17–66.71) to short term choices (M = 39.59, 95% CI 33.29–45.83) compared to positive emotional recall and positive post-recall mood modulation's undifferentiated long term (M = 52.10, 95% CI 45.00–59.20) and short term choices (M = 47.90, 95% CI 40.80–55.00, please see Table 1 for the results).

**Table 1:** Effect of emotional recall, recall specificity, and post-recall mood regulation on long-term and frequency-based decision making in an all-male sample.

**Intertemporal decision making**	***S.S*.**	***F*_(1, 63)_**	***P***	***ηp* ^2^**
DV: Decks (A′+ B′ vs. C′+ D′)	5,876.22	17.452	0.000	0.22
IV: Recall (positive vs. negative)	21.985	0.065	0.799	0.01
IV: Recall-specificity (specific vs. overgeneralized)	25.86	0.077	0.783	0.01
IV: Post-recall mood (regulation vs. no-regulation)	0.190	0.001	0.981	0.00
Recall × Recall-specificity	159.49	0.474	0.494	0.01
Recall × post-recall mood	2,249.68	6.681	0.012	0.10
Recall-specificity × post-recall mood	752.72	2.24	0.14	0.10
Recall × Recall-specificity × post-recall mood	91.63	0.272	0.60	0.00
**Frequency-based decision making**				
DV: Decks (B′+ D′ vs. A′+ C′)	2,217.19	6.27	0.015	0.09
IV: Recall (positive vs. negative)	2,735.09	7.73	0.007	0.11
IV: Recall-specificity (specific vs. overgeneralized)	2,468.93	6.98	0.010	0.10
IV: Post-recall mood (regulation vs. no-regulation)	540.09	1.527	0.221	0.02
Recall × Recall-specificity	17.13	0.048	0.827	0.001
Recall × post-recall mood	58.29	0.165	0.686	0.003
Recall-specificity × post-recall mood	878.37	2.483	0.120	0.038
Recall × Recall-specificity × post-recall mood	67.06	0.190	0.665	0.003

Next, the effect of emotional recall, recall specificity, and post-recall mood regulation was assessed on frequency-based decision-making, and the results showed that the main effect of frequency-based deck choices was significant; participants made more choices from the infrequent punishment deck (M = 54.10, 95% CI 50.83–57.38) compared to frequent punishments decks (M = 45.90, 95% CI 42.62–49.17). The effect of emotional recall was significant; participants who recalled negative emotion chose equally from infrequent punishment decks (M = 49.55, 95% CI 45.15–53.94) and frequent punishment decks (M = 50.45, 95% CI 46.06–54.85), whereas those who recalled positive emotion chose more from the infrequent punishment decks (M = 58.66, 95% CI 53.81–63.51) than the frequent punishment decks (M = 41.34, 95% CI 36.49–46.19). The effect of recall specificity was significant; participants with recall specificity drew equally from the frequent punishment decks (M = 50.23, 95% CI 45.56–54.89) and infrequent punishment decks (M = 49.77, 95% CI 45.11–54.44), whereas poor recall specificity (over-generalized recall) showed preference for infrequent punishment decks (M = 58.43, 95% CI 53.83–63.03) compared to frequent punishment decks (M = 41.57, 95% CI 36.97–46.17).

Two hierarchical regressions were used to compare the effects of emotional recall, recall specificity, and post-recall mood regulation on deck choices (intertemporal and frequency-based choices, please see Table 2). The first hierarchical regression addressed z-transformed long-term deck choices (dependent variable: long-term good decks C′ and D′) with emotional recall, recall specificity, and post-recall mood entered block-wise. The results indicated that block-wise addition of emotional recall, recall specificity, and post-recall mood regulation failed to account for long-term decision choices (decks C′ + D′), as there was no significant change in R-square values, and the model, as well as the predictors, remained non-significant (*p* > 0.05). Adding interaction terms showed no significant change for decks C′ + D′. The main effects of recall, recall specificity, and post-recall mood are reported; two-way interaction terms of recall × recall specificity, recall × post-recall mood, and the three-way interaction term of recall × recall specificity × post-recall mood are excluded.

**Table 2:** Hierarchical regression showing emotional recall (model 1), post-recall mood regulation (model 2), recall specificity (model 3) accounting for IGT decision–making based on intertemporal and frequency-based choices of an all-male sample (*N* = 71).

**Model**	**Predictors**	**DV** = **intertemporal deck (C**^**′**^+ **D**^**′**^**)**			**DV** = **frequency deck (B**^**′**^+ **D**^**′**^**)**		
		***B***	***SE***	**BCa 95% CI**	***B***	***SE***	**BCa 95% CI**
Model 1	Recall	0.003	0.24	[−0.46–0.48]	0.63^**^	0.23	[0.18–1.10]
		*R^2^* = 0.00, *F*Δ = 0.00, *F*_(1, 69)_ = 0.00			*R^2^* = 0.10^**^, *F*Δ = 7.62, *F* (1,69) = 7.62^**^		
Model 2	Recall	0.007	0.24	[−0.46–0.49]	0.59^**^	0.22	[0.13–1.04]
	Recall specificity	0.062	0.24	[−0.43–0.51]	−0.60^**^	0.22	[−1.00–−0.16]
		*R^2^* = 0.00, *F*Δ = 0.07, *F*_(2, 68)_ = 0.03			*R^2^* = 0.19^**^, *F*Δ = 7.49, *F*_(2, 68)_ = 7.91^**^		
Model 3	Recall	0.014	0.25	[−0.47–0.52]	0.56^**^	0.22	[0.11–1.01]
	Recall specificity	0.065	0.25	[−0.43–0.52]	−0.61^**^	0.21	[−1.03–−0.19]
	Post-recall mood	0.062	0.24	[−0.40 −0.55]	−0.28	0.21	[−0.67–0.14]
		*R^2^* = 0.00, *F*Δ = 0.06, *F*_(3, 67)_ = 0.04			*R^2^* = 0.21, *F*Δ = 1.68, *F*_(3, 67)_ = 5.89^**^		

Predictors were entered in three blocks: emotional recall (positive recall = 1) followed, recall specificity (specificity coded = 1), and post-recall mood (positive mood regulation = 1). F values and change in F values, BCa values bootstrapped (2000) 95% confidence interval, level of significance indicated as ‘^**^' = *p* < 0.01. Durbin–Watson values for intertemporal and frequency–based choices (decks B′ + D′) was within the recommended range of 1–3, suggesting independence of errors.

The second hierarchical regression addressed frequency-based deck choices (dependent variable: z transformed infrequent punishment decks B′ and D′). The results indicated that emotional recall accounted for infrequent punishment decks (10% variance), and recall of positive emotion (coded as 1) significantly accounted for the choice of infrequent punishment decks. Adding recall specificity increased the explanatory power as model 2; a positive recall was associated with a higher preference for infrequent punishment decks, and recall specificity was negatively associated with infrequent punishment decks; the variables accounted for 19% of the choices of infrequent punishment decks. Adding post-recall mood regulation in model 3 did not increase the explanatory power (model *p* > 0.05). However, emotional recall and recall specificity independently accounted for infrequent punishment decks, and change in R-square after adding post-recall mood was non-significant (*p* > 0.05). Post-recall mood was not a significant predictor of infrequent punishment decks (*p* > 0.05). Adding two-way interactions of recall x specificity, and recall x post-recall mood showed significant F change (*p* = 0.017) in preference for infrequent punishment decks (B′ + D′). However, the models were non-significant (all *p* > 0.05).

### Discussion

It was believed that affect control in emotional recall in recall specificity and post-recall mood regulation will facilitate long-term decision-making in the Iowa Gambling Task. Participants made more long-term decision-making drawing from safe decks compared to the short-term risky decks; valence of emotional recall did not influence long-term decision-making; however, only the interaction of emotional recall and post-recall mood regulation was significant—emotional recall and incongruency of post-recall mood facilitated long term decision making such that negative recall and post-recall positive mood was associated with more long term decisions in the task. The results support the hypothesis that negative mood in the presence of self-regulation via positive recall might reflect greater affect control in emotional recall; affect control of negative emotional recall demands greater cognitive control than affect control of positive emotional recall (Ochsner and Gross, 2005).

The incongruence between negative recall and post-recall positive mood implicated affect control might reflect successful inhibition of negative affect and flexibly switching between negative and positive affect; this adaptive self-regulatory capacity of exercising affect control in emotional recall might be facilitative for inhibiting the impulse to choose short term rewards and navigate choices toward long-term decision making via affect control over the positive and negative affect generated by the intertemporal reward schedule of the task. Results align with other observations where regulation of negative emotions (sadness, disgust) benefited long-term decision-making (e.g., Heilman et al., 2010; Bollon and Bagneux, 2013). The present result suggests that affect control of negative emotion via post-recall positive mood increases the choice of cognitively demanding long-term decision-making. The main effects of the valence of recall, the restorative post-recall mood regulation, and recall specificity were non-significant, indicating that valence of recall (positive/negative emotional recall), positive post-recall mood regulation (post-recall positive mood/post-recall non-positive mood), and recall specificity (specific/overgeneralized recall) had no independent effects on long term decision making.

The results for frequency-based decision-making indicated the main effect on deck choices: infrequent punishments were preferred over frequent punishment decks, emotional recall had an effect on frequency-based decisions as negative emotional recall was associated with indifference to frequency-based decision-making because they chose equally from the frequent and infrequent punishment decks, whereas positive emotional recall was associated with preference for infrequent punishment decks. There was an effect of recall specificity on frequency-based decision-making; those with recall specificity were indifferent and chose equally from frequent and infrequent punishment decks, whereas poor recall specificity/overgeneralized recall was associated with a preference for infrequent punishment decks. Results indicate that positive recall invites low self-regulation and poor recall specificity, indicating poor cognitive processing; both independently indicated low engagement of cognitive resources (Hawthorne and Pierce, 2015) and high engagement of emotion-based information processing indicative of sensitivity to frequency of rewards (Stocco et al., 2009). Recall specificity can be observed independent of emotion valence (Guler and Mackovichova, 2019), explaining emotional recall and recall specificity had independent effects on the frequency attribute of decision-making. Post-recall mood regulation did not affect frequency-based decision-making, indicating that self-regulatory control over mood might be unrelated to emotion-based information processing reflected in frequency decision-making. Because post-recall mood regulation was observed to facilitate long-term decision-making, these results align with the dual-process account of decision-making in the task (Stocco et al., 2009), wherein post-recall mood facilitated cognitive-based long-term decision-making but remained unrelated to emotion-based frequency choices. The interactions of emotional recall, recall specificity, and post-recall mood regulation did not impact frequency-based decision-making.

In support of the analysis of variance, where interpretation of complex two-way and three-way interactions poses a challenge, two hierarchical regressions were employed, which enabled comparing independent effect and interaction effects of emotional recall, recall specificity, and post-recall mood regulation in a block-wise manner to understand the role of factor and their interactions (negative recall, recall specificity, and post recall mood regulation) in decision making. It also enabled deriving inference using bootstrapped sampling distribution (Davidson et al., 2000) to provide more robust insights in case of a modest sample size. Results for the long-term decision-making (long-term choices C' + D') showed that model 1 (emotional recall) and model 2 (recall specificity) were non-significant. Model 3 (post-recall mood regulation) failed to account for long-term decision-making, indicating that the inference drawn from the analysis of variance for long-term decision-making might be variable and dependent on the sample size. On the other hand, the results for regression accounting for frequency-based decision-making indicated that positive emotional recall was associated with a preference for infrequent punishment decks, significantly accounting for 10% of infrequent punishment deck choices (decks B' + D'). Positive emotional recall was associated with the less-demanding frequency-based preference. In a previous study, the emotion that triggers optimism and certainty engages frequency-based heuristic processing and a risky preference for infrequent punishment deck (Iyilikci and Amado, 2018). Because positive recall places less demand on cognitive resources compared to negative recall, it might have been associated with the lesser demanding frequency attribute of decision-making. Adding recall specificity increased the explanatory power and accounted for 19% of infrequent punishment decks, and recall specificity was associated with fewer choices of infrequent punishment decks. Adding post-recall mood did not affect the mood, indicating that post-recall mood regulation did not influence frequency-based choices.

Compared to cognitively demanding long-term decision-making, the poor cognitive engagement reflected in frequency-based choices showed stable results across the two statistical approaches—positive recall was associated with poor cognitive engagement and high preference for infrequent punishment decks (B′ + D′), recall specificity indicated cognitive control that possibly helped inhibit choice of infrequent punishment decks. Low cognitive demands of positive emotional recall or poor recall specificity engage heuristic, low-demanding choice attribute of frequency rather than a more demanding intertemporal attribute.

## General discussion

This investigation examined an assumption of the somatic marker hypothesis; it tested whether affect control might be a potential mechanism for emotional recall facilitating long-term decision-making in the Iowa gambling task. The results of Experiment 1, sequential recall of two polarized emotion events were used, and it indicated that affect control in emotional recall (use of executive control to switch from negative to positive affect) facilitates long-term decision making, specifically the emotional recall sequence of negative-positive emotional recall was associated with long-term decision making. In contrast, frequency-based decision-making showed no effect from emotional recall sequence. The results of experiment 2 examined recall specificity and post-recall mood regulation as measures of affect control in emotional recall that might facilitate long-term decision-making. In support of the regulatory control in affect states, negative emotional recall in the presence of post-recall restorative positive mood was associated with more long-term decisions.

Conversely, positive emotional recall and poor recall specificity were associated with frequency-based preference for infrequent punishment decks. Engaging low affect control (recall of positive emotion and poor recall specificity) might be linked with less cognitively demanding frequency-based choice. Of the two attributes that differ in cognitive demands, the more demanding intertemporal decision-making and the less demanding frequency-based decision-making in the Iowa Gambling Task, supportive analysis using hierarchical regression with bootstrapping for sample distribution supported that emotional recall and recall specificity account for frequency-based choices. In contrast, post-recall mood regulation does not relate to low cognitive engagement observed in frequency-based choices.

The results align with the double-dissociable of the two attributes of decision-making in the IGT (Stocco et al., 2009) and, to some extent, with the dual-process theory that suggests the disproportionate influence of negative affect on decision-making (e.g., Rozin and Royzman, 2001; Kahneman and Tversky, 2013). The results provide a potential mechanism through which affect control engaged in emotional recall might be a cognition-intensive process of using executive control for affect regulation (Ochsner and Gross, 2005), and this self-regulation facilitates inhibition of short-term temptations in favor of long-term decision-making. Although the ability to induce emotion from the recall of an emotional event (secondary inducer) is critical for decision-making (Bechara et al., 2000a), unregulated affect induced via emotional recall might interfere with long-term decisions in the IGT (Bechara and Damasio, 2005). Affect control is not only self-regulatory but also benefits adaptive long-term decision-making; poor affect control (e.g., positive emotional recall or poor recall specificity) reflects low deployment of cognitive control and maladaptive preference for least demanding frequency-based decision-making.

These results need to be interpreted within several limitations. Although the choice of analysis was aimed at addressing an unbalanced group in Experiment 1, an equal sample size for the emotional recall sequence groups would have enabled a robust between-group comparison. The small sample size in Experiment 2 might be a limitation; an attempt to remedy it was to use hierarchical regression to compare affect control in recall, specificity, and post-recall mood regulation. It was observed that the results for frequency-based decision-making remained stable with bootstrapped sampling applied to the small sample size. When post-recall mood regulation was examined in Experiment 2 for incongruency of emotional recall and post-recall mood, the absence of baseline mood measure prior to the emotional recall was not taken. Although the mood measure assessed the mood prevailing over the last few days, the mood measure at the start of the experimental protocol would have better controlled post-recall mood alteration. Free recall and writing a detailed description of emotion decision was used to facilitate emotional recall, and subjective ratings of the emotional recall provided a self-rated strength of emotional recall; however, future studies could measure physiological and neural changes that accompany affect and affect control induced by emotional recall (e.g., skin conductance response, heart rate variability, hemodynamic response); the present study had no funding for equipment to record physiological alteration. Although participation was not incentivized by payment or course credits, the study protocol ensured that the participants performed as per the instructions and used self-set goals to elicit task motivation.

Further, balancing the groups in terms of gender could not be done due to female underrepresentation in engineering institutions. Despite several limitations, the present results helped explore a potential mechanism through which control over affect in emotional recall might facilitate long-term decision-making; preliminary evidence indicates that affect control over emotional recall, particularly self-regulatory control in negative emotional recall, might improve adaptive long-term decision-making. The somatic marker hypothesis has contributed immensely to the understanding of emotions as a somatic affective influence on decision making; Damasio's original conceptualization of emotional recall and affect as a simple binary, unipolar construct navigating decision making through choices that are “good” or “bad” now accommodates affective experiences coded as ambivalent, engaging counter-factual thoughts (Vaccaro et al., 2020). The next step might be to examine cognitive processes deployed to generate, re-generate, regulate, maintain, alter, and inhibit affective experiences of the past in service of future decision-making. Investigations of neuroanatomical correlates of affect control engaged in emotional recall and decision-making, in terms of the connectivity between amygdala-hippocampal and ventromedial and dorsolateral subregions of the prefrontal cortex, might add insights to affect dysregulation in negative emotional recall and short-term, impulsive decision making in depression (e.g., poor affect control in negative emotional recall might lead to a decision to end life based on short-term thinking). Although the present work is exploratory and has several limitations (sample size, baseline mood measure), it explores a key assumption of the somatic marker hypothesis, specifically the mechanism through which somatic/affective influence induced in emotional recall might facilitate decision-making. The results might be preliminary but essential in exploring the role of affect control via more than one method, to test whether the extent of self-regulatory control might determine the nature of the affective influence on decision-making. It presents a possibility to revive the Jamesian argument of how lower-order somatic/autonomic regulation contributes to cognitive flexibility and inhibitory control to facilitate higher-order decision-making.

## Data availability statement

The raw data supporting the conclusions of this article will be made available by the authors, without undue reservation.

## Ethics statement

The studies involving humans were approved by Institute Ethics Committee (IEC), Indian Institute of Technology Delhi. The studies were conducted in accordance with the local legislation and institutional requirements. The participants provided their written informed consent to participate in this study.

## Author contributions

The author confirms being the sole contributor of this work and has approved it for publication.

## Appendix A

The instructions for the Iowa Gambling Task were as follows:

“In front of you on the screen, there are four decks of cards: A′, B′, C′, and D′. When we begin the game, I want you to select one card at a time by clicking on a card from any deck. Each time you select a card, the computer will tell you that you have won some money. I don't know how much money you will win. You will find this out as you go along. Every time you win, the green bar at the top of the screen gets bigger. Every so often, when you click on a card, the computer will tell you that you have won some money as usual, but it will also say that you have lost some money. I don't know when you will lose or by how much. You will find out as you go along. Every time you lose, the green bar at the top of the screen gets smaller. You are absolutely free to switch from one deck to another at any time, and as often as you wish. The goal of the game is to win as much money as possible and to avoid losing as much money as possible. You won't know when the game will end. Simply keep on playing until the computer stops. You will have $2,000 of credit, shown by the green bar, at the start of the game. The only hint I can give you, which is the most important thing to note, is this: out of these four decks of cards, some are worse than others. To win, you should try to stay away from bad decks. No matter how much you find yourself losing, you can still win the game if you avoid the bad decks. Moreover, the computer does not change the position of the decks once the game begins. It does not make you lose at random, or make you lose money based on the last card you picked.”
